# Association Between Self-Perceived Oral Health, Stress, and Oral Symptoms in South Korean University Students: A Cross-Sectional Study

**DOI:** 10.3390/healthcare13090984

**Published:** 2025-04-24

**Authors:** Jinhyoung Jeong, Wooyoung Jeong, Yuyeon Jung

**Affiliations:** 1 Department of Biomedical Management, College of Medical Convergence, Catholic Kwandong University, 24, Beomil-ro 579beon-gil, Gangneung-si 25601, Republic of Korea; wlsgud0201@cku.ac.kr; 2 Department of Biomedical Sciences, College of Medical Convergence, Catholic Kwandong University, 24, Beomil-ro 579beon-gil, Gangneung-si 25601, Republic of Korea; wyjeong@cku.ac.kr; 3 Department of Dental Hygiene, College of Medical Science, Konyang University, 158 Gwanjeodong-ro, Seo-gu, Daejeon 35365, Republic of Korea

**Keywords:** oral health, university students, lifestyle, stress, self-perceived oral health

## Abstract

**Background/Objectives:** Self-perceived oral health is influenced by clinical and subjective oral factors, socioeconomic status, stress, and oral health behaviors. However, few studies have examined its association with dry mouth, salivary viscosity, and lifestyle factors. This study investigated the impact of self-perceived oral health and stress levels on subjective oral symptoms and lifestyle factors. **Methods:** A total of 644 university students participated. Self-perceived oral health was subjectively evaluated using a single item, and stress levels were measured using the Korean version of the Perceived Stress Scale-10 (PSS-10), which has been validated for reliability and validity. Chi-square tests identified differences in self-perceived oral health and stress levels based on subjective oral symptoms and lifestyle factors. Logistic regression assessed the effects of tooth brushing frequency, periodontal disease diagnosis, dry mouth, and gingival bleeding on self-perceived oral health. **Results:** Subjective oral symptoms significantly associated with self-perceived oral health included gingival bleeding, dry mouth, and salivary viscosity; lifestyle factors included tooth brushing frequency and beverage consumption. Frequent tooth brushing increased the likelihood of perceiving oral health as good (OR = 1.363, *p* = 0.030), while dry mouth (OR = 0.576, *p* = 0.001) and gingival bleeding (OR = 0.597, *p* = 0.003) reduced it. **Conclusions:** This study’s results showed that gingival tooth brushing frequency and subjective oral symptoms significantly impacted self-perceived oral health. Therefore, effective educational programs should be implemented to promote self-perceived oral health awareness and oral health maintenance.

## 1. Introduction

Oral health is a multifaceted and essential component of overall health, allowing us to perform daily activities, such as speaking, smiling, tasting, and chewing [1]. The World Health Organization defines oral health as the absence of conditions that limit a person’s psychosocial well-being and capacity [2]. Recently, there have been efforts to expand the concept of oral health. Fejerskov presented oral health not merely as the absence of disease, but as a multidimensional concept encompassing personal autonomy, social participation, and emotional well-being. This approach emphasizes that oral health is closely related to the quality of life and psychosocial functioning, highlighting the need to understand oral health from a more integrated perspective [3]. Maintaining oral health is crucial because it can improve mental and overall health [4]. Factors that determine oral health include physiological functions, psychosocial functions, disease conditions, environmental factors, and self-perceived oral health [5].

Self-perceived oral health is defined as a subjective evaluation in which individuals assess their overall oral health status, including experiences and perceptions related to the functional condition, pain, and aesthetics of the oral cavity [6]. According to previous studies, self-perceived oral health is associated with clinical and subjective oral factors, socioeconomic status, perceived stress, and oral health behaviors [7]. In particular, its strong correlation with clinical indicators suggests that self-perceived oral health may serve as a valuable reflection of an individual’s actual oral condition [8]. Clinical factors include dental caries, tooth loss, and gingival bleeding; subjective factors are related to general health status and oral pain [7]. Additionally, clinical and subjective oral factors such as tooth loss, gingival bleeding, and pain influence perceptions of one’s oral health, well-being, and quality of life [7]. Therefore, to maintain self-perceived oral health, related factors must be controlled and managed.

Several countries are examining self-perceived oral health, including China and Japan [9,10]. However, identifying the determinants of self-perceived oral health is crucial because the factors associated with them vary by race [7]. Therefore, further studies on self-perceived oral health and overall oral health in South Korea are needed. According to previous Korean studies, the stress levels of university students have been shown to negatively affect their self-perceived oral health status [11]. Furthermore, negative perceptions of oral health among university students were associated with lower self-efficacy and increased fear of forming social relationships [12]. These findings suggest that Korean university students are a population whose perception of oral health may be influenced not only by physical and psychological changes but also by social pressures related to academics and employment. Specifically, university students are young adults undergoing dynamic growth and may experience changes in their health, social psychology, lifestyle, and behavior [9]. Additionally, many university students are living away from home for the first time and are responsible for their health, lifestyle, and behavior; poor health habits can significantly impact their self-perceived oral health [7,13]. Previous studies on oral health have primarily focused on clinical perspectives or specific age groups, with relatively limited research targeting university students. This study aims to address these gaps by exploring the relationships between self-perceived oral health awareness, stress, and lifestyle habits among university students.

Therefore, the following hypotheses were established in this study. First, individuals with a more positive self-perception of oral health will have a lower incidence of subjective oral symptoms. Second, lower stress levels will be associated with healthier lifestyle habits. Third, oral symptoms will have a significant impact on self-perceived oral health.

This study aimed to analyze the factors influencing self-perceived oral health among university students in South Korea. In particular, it examined the associations between subjective oral symptoms, lifestyle habits, and stress levels with self-perceived oral health, with the goal of providing foundational data to support improvements in oral health awareness among university students.

## 2. Materials and Methods

### 2.1. Study Design and Ethical Considerations

This study was a cross-sectional survey conducted among university students enrolled in universities located in Gangwon and Daegu, South Korea. Data were collected using a self-administered structured questionnaire distributed online via Google Forms. This study complied with the ethical principles outlined in the Declaration of Helsinki (2008) and was reported in accordance with the STROBE guidelines.

### 2.2. Participant Recruitment and Sample Size Calculation

A non-probabilistic sampling method was used to recruit participants. The minimum required sample size was calculated to be 136 using G*Power 3.1.9.7 software, based on a significance level (α) of 0.05, an effect size of 0.15, and a statistical power of 0.90. A total of 660 university students participated in the survey. After excluding 16 responses deemed insincere based on logical inconsistency between items, 644 responses were included in the final analysis (57 males and 587 females).

### 2.3. Survey Composition and Variables

This study was conducted in accordance with established guidelines for reporting medical investigations [14]. The questionnaire was created by modifying items from earlier surveys [15,16]. The questionnaire comprised questions regarding general characteristics, self-perceived oral health, subjective oral symptoms, lifestyle, stress levels, and periodontal disease diagnosis. General characteristics included gender (male, female) and grade (1st, 2nd, 3rd, and 4th grades). Self-perceived oral health was classified as good and bad, and subjective oral symptoms were classified according to gingival bleeding, the presence of dry mouth, and the viscosity of saliva (very low, low, high, very high). Lifestyle factors included the average frequency of daily tooth brushing (one to two times, three times, four or more times), smoking status (yes/no), average weekly frequency of consumption of beverages such as soda and ale (no beverages consumed, one to two times, three to four time, five or more times), average daily sleep duration (5 h or less, 6 h or less, 7 h or less, 8 h or more) and oral hygiene aids used (not used, interdental brush, dental floss, mouthwash, water pick or tongue cleaner). The items related to self-perceived oral health, subjective oral symptoms, and lifestyle habits were developed with reference to the questionnaire items from the Korea National Health and Nutrition Examination Survey (KNHANES), based on indicators that have been validated for reliability and validity. The stress level assessment was measured using the Korean version of Cohen’s Perceived Stress Scale-10 (PSS-10) [17,18], and the response results were classified into four categories (very low, low, high, very high). The presence of periodontal disease was based on a clinically validated self-reported measure. The questions were “Have you ever been told by a dental professional that you lost bone around your teeth?” the range of answers were (Yes, No) [19].

### 2.4. Data Collection Procedure

After explaining the purpose and procedures of our study, participants provided voluntary consent and completed the online questionnaire. To ensure response validity, responses containing logical inconsistencies between items were identified and excluded from the analysis as insincere.

### 2.5. Statistical Analysis

The collected data were analyzed using SPSS Statistics version 22.

Chi-square tests were performed to identify differences in subjective oral symptoms and lifestyle factors according to self-perceived oral health and stress levels.

A one-way ANOVA was conducted to examine mean differences in lifestyle behaviors (tooth brushing frequency, smoking status, beverage intake frequency, sleep duration) and subjective oral symptoms (salivary viscosity, dry mouth, gingival bleeding) across stress level groups. Tukey’s post hoc test was used for pairwise comparisons. To ensure consistency in the interpretation of variables, gingival bleeding, dry mouth, and smoking status were reverse-coded so that higher values indicate greater severity or frequency of each characteristic. This allowed for higher mean values to be interpreted as reflecting poorer health conditions, thereby facilitating clearer visual and statistical comparisons.

Logistic regression analysis was used to determine the effects of tooth brushing frequency, periodontal disease diagnosis, and subjective oral symptoms (gingival bleeding and dry mouth) on self-perceived oral health. To further explore the relationships among stress, lifestyle factors, and subjective oral symptoms, Pearson correlation analysis was conducted.

The goodness-of-fit of the logistic regression model was assessed using Nagelkerke’s R^2^ and the Hosmer–Lemeshow test. The Nagelkerke R^2^ value of the model was 0.061, indicating relatively low explanatory power. However, the Hosmer–Lemeshow test result (*p* = 0.798) suggested that the model had a good fit to the observed data. Additionally, because logistic regression does not require assumptions of normality, linearity, or homogeneity of variance–covariance matrices (unlike discriminant or multiple regression analyses), a separate normality test was not conducted. The level of statistical significance was set at 0.05.

## 3. Results

### 3.1. Differences in Subjective Oral Symptoms and Lifestyle According to Self-Perceived Oral Health

A chi-square test was conducted to verify whether there were significant differences in subjective oral symptoms and lifestyle factors according to the participants’ self-perception of oral health (Table 1). Among the participants, the number of those who perceived their oral cavity as healthy significantly exceeded the number of those who perceived it as unhealthy, and they reported no gingival bleeding (66.4%, *p* < 0.001), no dry mouth (66.0%, *p* < 0.001), or meager saliva viscosity. (63.3%, *p* = 0.035). Additionally, the proportion of participants who perceived their oral health as healthy was significantly higher when they brushed their teeth three times (64.0%, *p* = 0.031) and when they did not consume any beverages (69.2%, *p* = 0.024) than when they perceived their oral cavity as unhealthy. However, there was no statistically significant difference in self-perceived oral health between smoking status and sleep duration.

### 3.2. Differences in Subjective Oral Symptoms and Lifestyle According to Stress Levels

A chi-square test was conducted to verify whether there were significant differences in subjective oral symptoms and lifestyle factors according to participants’ stress levels (Table 2). Regarding subjective oral symptom factors, gingival bleeding (55.1%, *p* = 0.002), no dry mouth (55.6%, *p* = 0.032), and very low salivary viscosity (54.3%, *p* = 0.002) were observed when the stress level was low. Regarding lifestyle factors, when the stress level was low, the respondents were non-smokers (53.9%, *p* < 0.001) and the average daily sleep duration was less than seven hours (59.3%, *p* = 0.032). However, there was no significant difference in stress levels between the average daily tooth brushing frequency and the average weekly frequency of beverage consumption.

### 3.3. Associations Between Stress Levels, Lifestyle Habits, and Oral Symptoms

**Figure 1:**
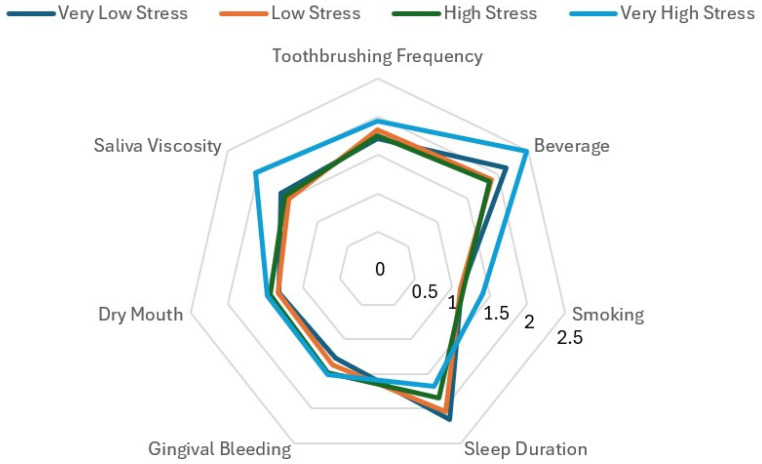
Association of Stress Levels with Oral Health Behaviors and Subjective Symptoms.

Figure 1 shows the differences in lifestyle habits and subjective oral symptoms according to stress levels. As stress levels increased, average daily sleep duration decreased, with the lowest mean observed in the “Very High Stress” group. Conversely, smoking status, beverage consumption frequency, and salivary viscosity showed the highest average values in the “Very High Stress” group. Additionally, dry mouth and gingival bleeding tended to increase with higher stress levels. These findings suggest that stress levels affect both lifestyle behaviors and oral symptoms, indicating that stress may influence oral health indirectly through behavioral factors.

### 3.4. Factors Affecting the Use of Oral Hygiene Aids

**Figure 2:**
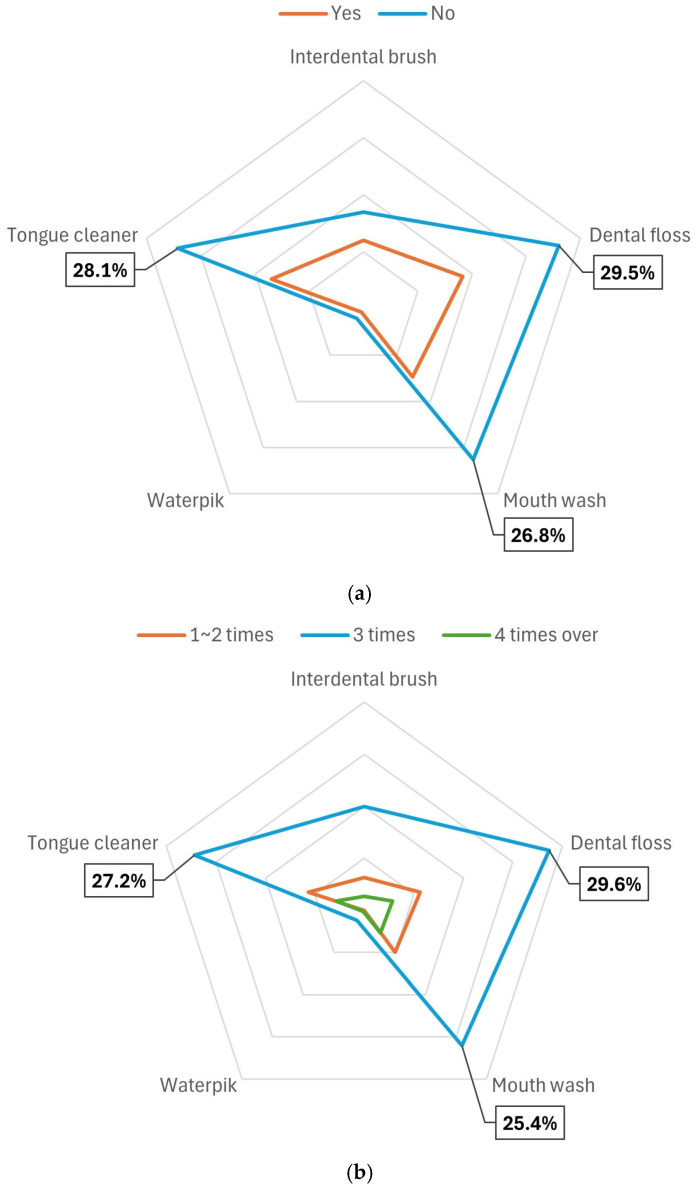

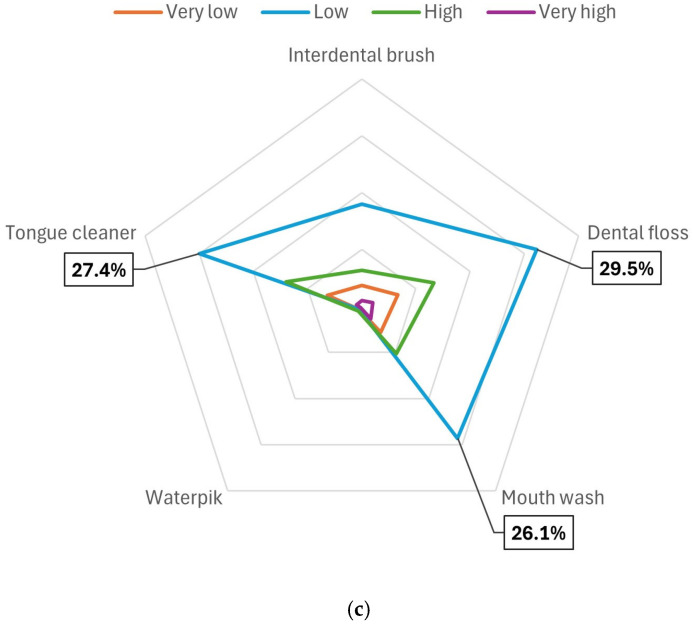
(**a**) Factors influencing oral hygiene aid use: gingival bleeding. (**b**) Factors influencing oral hygiene aid use: tooth brushing frequency. (**c**) Factors influencing oral hygiene aid use: stress level.

Figure 2 shows the differences in the use of oral hygiene aids according to gingival bleeding, average daily tooth brushing frequency, and stress levels among individuals who perceived their oral health as healthy. The rate of use of oral hygiene aids was highest when there was no gingival bleeding, when the average daily tooth brushing frequency was three times, and when stress levels were low. Dental flosses, tongue cleaners, and mouthwashes were the most frequently used oral hygiene aids.

### 3.5. Factors Affecting Self-Perceived Oral Health

A logistic regression analysis was conducted to determine the impact of tooth brushing frequency, periodontal disease diagnosis, dry mouth, and gingival bleeding on self-perceived oral health (Table 3). During the analysis, variables whose association was not appropriate or statistically significant were excluded from the final model. Periodontal disease diagnosis has been widely examined in previous studies as a key factor closely associated with self-perceived oral health. Therefore, to maintain consistency with existing research and enhance comparability, this variable was included in the final model. The analysis revealed that individuals who brushed their teeth more frequently were more likely to perceive their oral health as good (OR = 1.363, 95% CI = 1.030–1.804, *p* = 0.030). In addition, individuals experiencing dry mouth (OR = 0.576, 95% CI = 0.413–0.804, *p* = 0.001) or gingival bleeding (OR = 0.597, 95% CI = 0.426–0.836, *p* = 0.003) were significantly less likely to perceive their oral health as good.

### 3.6. Correlations Among Key Variables

Table 4 presents the results of a Pearson correlation analysis between stress levels, oral symptoms, and lifestyle factors.

Stress levels showed significant positive correlations with gingival bleeding (r = 0.148, *p* < 0.001), dry mouth (r = 0.106, *p* = 0.007), saliva viscosity (r = 0.078, *p* = 0.047), average weekly beverage consumption frequency (r = 0.078, *p* = 0.049), and sleep duration (r = 0.143, *p* < 0.001). These findings suggest that higher levels of stress are associated with a worsening of oral symptoms and health behaviors.

Meanwhile, smoking status showed a significant negative correlation with stress (r = −0.134, *p* = 0.001), indicating that individuals with higher stress levels tend to be more likely to smoke.

## 4. Discussion

Self-perceived oral health is used in epidemiological studies because, in addition to disease, it is influenced by functional capacity, pain, aesthetics, and psychosocial factors [20]. The key factors associated with self-perceived oral health include clinical and subjective oral health [7]. However, limited studies examine whether self-perceived oral health is influenced by dry mouth, salivary viscosity, or lifestyle factors. Investigating these factors is crucial to improve self-perceived oral health. Therefore, this study investigated factors related to self-perceived oral health among South Korean university students.

The U.S. Centers for Disease Control and Prevention (CDC) defined oral health as the health status of teeth, gingiva, and oral facial systems and reported that diseases affecting oral health include dental caries, periodontal disease, oral cancer, and tooth loss [21]. Periodontal disease is an inflammatory disease that affects the soft and hard tissues that support teeth and causes gingival bleeding, pain, redness, swelling, tooth agitation, and loss [22,23]. When bleeding or pain occurs in the gingiva, it affects speech and pronunciation, causing difficulties in social interaction and negatively affects psychological health through feelings of guilt, discomfort, or lack of care [24]. According to Luchi et al.’s study, people experiencing periodontal disease, dry mouth, cavities, and tooth loss often perceived their oral cavity as unhealthy [25]. This aligns with our results that people who perceived their oral health as healthy did not have gingival bleeding, pain, or dry mouth compared with people who perceived their oral health as unhealthy and had minimal saliva viscosity.

The findings of this study are consistent with previous research conducted in Japan, which found that oral health behaviors such as tooth brushing frequency and the use of oral hygiene aids were significantly associated with self-perceived oral health [10]. Additionally, a study from China also reported that university students’ oral health knowledge, attitudes, and behaviors were closely related to their self-perceived oral health [9,26]. These international research findings support the importance of fostering healthy oral habits to enhance oral health awareness among university student populations. Previous studies have reported that self-perceived oral health is associated with the development of good oral health behaviors [27]. This aligns with our results, which showed that self-perceived oral health is significantly related to lifestyle. Lifestyle factors related to self-perceived oral health included the average daily tooth brushing frequency and average weekly frequency of beverage consumption. The group that perceived their oral health as healthy brushed their teeth an average of three times per day (64.0%). People consider brushing their teeth a fundamental self-care behavior for maintaining oral health [28]. Tooth brushing helps prevent and relieve dental caries and dry mouth by removing plaque from teeth and temporarily increasing saliva secretion [29,30]. Therefore, many people believe that brushing their teeth helps maintain overall physical hygiene and health, and they expect that brushing their teeth will make their mouths healthier [31]. This finding supports that self-perceived oral health is influenced by the frequency of tooth brushing. Furthermore, studies have reported that individuals who undergo regular dental check-ups tend to have a more positive self-perception of their oral health as they gain insights into their oral health status and learn effective management strategies [31,32]. These findings suggest that a more positive self-perception of oral health is associated with a higher likelihood of practicing proper oral health behaviors.

Moreover, self-perceived oral health was associated with beverage consumption habits. Specifically, Korean college students use energy drinks to overcome sleep deprivation when studying for exams or completing projects [33]. Energy drinks are used to improve mental and physical performance and have gained global popularity over the years, making them the fastest-growing drink in the global beverage market [34]. However, these drinks contain excess amounts of sugar and are highly acidic. Thus, they pose a danger to oral health due to the risk of leading to dental caries and tooth erosion. Furthermore, their excessive consumption can increase stress levels among university students with sleep disorders [35,36,37]. Recent studies have shown that higher levels of psychological stress are associated with an increased consumption of sugar-sweetened beverages [38,39]. This behavior is related to the tendency to consume functional or sugary drinks excessively as a coping mechanism for stress, indicating that beverage consumption is closely linked to an individual’s psychological state [40]. Therefore, their consumption has emerged as a global public health problem [34]. This shows how subjective oral symptoms and incorrect lifestyle habits significantly impact oral health, and the need for forming a positive self-perception of oral health by practicing a healthy lifestyle.

University students in South Korea are under considerable stress due to academics, pressure to succeed, competition, and financial burdens [41,42]. University students under significant stress change their eating and lifestyle, negatively affecting their emotions and health [43,44]. Therefore, we investigated and analyzed the relationships among stress, subjective oral symptoms, and lifestyle to determine the factors affecting university students’ health. Stress affects health directly and indirectly through changes in autonomic and neuroendocrine responses and health behaviors [45]. Specifically, if stress is not relieved, various problems arise. According to Stankeviciene’s study on stress among university students, high stress levels lead to unhealthy behaviors, such as incorrect eating habits, smoking, and drinking, leading to failure to control body homeostasis [43,46]. Failure to maintain homeostasis causes weakening of the periodontal tissue and decreased saliva secretion due to local tissue destruction in the oral cavity [43,47]. When saliva secretion decreases, complications such as dry mouth, dental caries, periodontal disease, oral burning, and oral pain occur [48,49,50]. Furthermore, they do not have antibacterial, mastication, digestion, taste, lubrication, or pH buffering effects, making it challenging to maintain oral homeostasis [51,52]. Psychological stress is one of the major factors contributing to the deterioration of oral health, and recent cross-sectional studies have reported that stress has a negative impact on periodontal health. Previous studies have also suggested that stress can alter immune responses and oral hygiene habits, thereby accelerating the progression of periodontal disease [53]. Furthermore, higher levels of psychological stress have been found to be significantly associated with increased periodontal disease indices [54,55]. These findings are consistent with the present study, which demonstrated that higher levels of psychological stress were associated with gingival bleeding—a key indicator of periodontal health. Therefore, stress must be controlled appropriately to promote and maintain oral health. This study’s results showed that stress was closely related to subjective oral symptoms and lifestyle. Studies on the stress levels of university students show that when their stress level is low, they do not experience gingival bleeding, pain, or dry mouth, and the viscosity of their saliva is low. Stress also affects smoking and sleep duration. Additionally, higher stress levels were associated with an increase in unhealthy lifestyle habits and negative oral symptoms, suggesting the need for both stress management and the adoption of healthy lifestyle practices. The correlation between stress and smoking has been investigated using various methods [56,57,58]. Cigarettes are perceived to calm the mind and lead to focused thoughts when stress levels are high [59]. Moreover, stress affects sleep duration and quality; the shorter the sleep duration, the more negatively the person reacts to daily stressors and events [60]. Blood pressure, heart rate, hormone secretion, immune function, cell repair, memory recovery, and cognitive function are all regulated during sleep [61]. Lack of sleep is also related to respiratory and cognitive function decline, memory loss, and weight gain, and negatively impacts university students’ academic and overall health [61]. Therefore, the U.S. National Sleep Foundation recommends that adults get seven to nine hours of sleep [62].

People with high stress levels may neglect oral hygiene behaviors, leading to negative outcomes [53]. Additionally, studies show that stress increases anxiety, significantly impacting oral health [63]. Conversely, people with less anxiety may have better lifestyle-related and oral hygiene behaviors [63]. Oral hygiene behaviors include regular toothbrushing, use of oral hygiene aids, and regular dental checkups; practicing these oral hygiene behaviors helps prevent dental caries and periodontal diseases [64]. Furthermore, it affected various factors, including gingival status, oral hygiene knowledge, and stress, aligning with this study [31,65,66,67]. Therefore, this study investigated the effects of gingival bleeding, average daily tooth brushing frequency, and stress levels on the use of oral hygiene aids according to self-perceived oral health. The results showed that the rate of use of oral hygiene aids was high when there was no gingival bleeding, when the average daily tooth brushing frequency was three times and when the stress level was low, especially when the rate of use of oral hygiene aids was high, in the order of dental flossing, tongue cleaning and gargling. Therefore, to maintain good oral health, it is critical to remove plaque daily, and dental floss or an interdental brush is recommended to reduce gingivitis and plaque compared with brushing alone [68,69,70]. Additionally, individuals who brush their teeth regularly use more oral hygiene aids. Consistent results have shown that people who perceived their oral health as healthy did not have gingival bleeding and frequently used dental floss. A previous study reported that those who brushed their teeth more than three times per day were 1.394 times more likely to use interdental care products than those who brushed their teeth twice [71]. Additionally, people with knowledge of oral health are more likely to have better self-care habits [72]. Therefore, ongoing oral health education is required to increase the likelihood of people practicing better oral care habits [64]. Additionally, government intervention must provide and manage innovative and efficient educational programs to develop self-administered oral health management habits to maintain and improve oral health.

Oral diseases are caused not only by children unfamiliar with dental care, but also by chronic diseases such as diabetes, high blood pressure, and osteoporosis; additionally, young and middle-aged people complain of periodontal diseases caused by stress [73]. Due to the chronic and cumulative nature of oral diseases, dental care expenses constitute a significant proportion of the total medical costs and continue to increase steadily each year; the 2020 results for common dental outpatient diseases indicated that “gingivitis and periodontal disease” affected 16.27 million people (31.4%), making it the most prevalent condition, followed by “dental caries,” which affected 6.13 million people (11.8%) [73]. Therefore, it is critical for university students to develop proper lifestyles as they begin adulthood [74,75]; this study aimed to provide fundamental data for developing an efficient and practicable educational program for oral health management.

### Limitations

This study has several important limitations. First, it was conducted as a non-probabilistic survey targeting university students in specific regions of South Korea, which limits the generalizability of the findings to the broader university student population. In particular, the sample had a significant gender imbalance, with 91% of respondents being female, which may indicate an overrepresentation of responses from one gender. This imbalance could have affected the interpretation of results, as gender differences in self-perceived oral health and stress levels may not have been adequately reflected.

Second, due to the cross-sectional design, causal relationships between variables could not be determined. The findings only suggest associations, and it remains unclear whether certain lifestyle behaviors or symptoms influence self-perceived oral health or vice versa.

Third, although this study included students from various academic fields and university types, differences in stress levels between students in medical and non-medical colleges may exist. Future research should consider stratified analyses based on university type or academic discipline.

Fourth, because this study focused solely on university students, the sample lacked diversity in age distribution. Additionally, given that most students are not engaged in economic activities, information on socioeconomic status was limited. These factors may have influenced the interpretation of the results.

These limitations may have impacted our study findings. Therefore, future research should aim for a more representative sample with diversity in gender and academic background and consider employing a longitudinal design for more refined analysis.

## 5. Conclusions

This study examined the effects of self-perceived oral health awareness and stress on subjective oral symptoms and lifestyle behaviors. Participants with a positive perception of their oral health experienced fewer symptoms, such as gingival bleeding and dry mouth, brushed their teeth more frequently, and showed a reduced intake of sugar-sweetened beverages. Similarly, lower stress levels were linked to fewer subjective oral symptoms and healthier behaviors, including non-smoking and adequate sleep duration. Furthermore, gingival bleeding, pain, and dry mouth significantly influenced self-perceived oral health awareness.

These findings underscore the need for educational strategies aimed at enhancing oral health awareness and stress management, with the goal of promoting healthier lifestyles among university students. Future research should explore the causal relationships between stress and oral health perception and investigate whether similar associations exist across different demographic or cultural groups.

## Figures and Tables

**Table 1:** Association between subjective oral symptoms and lifestyle factors according to self-perceived oral health.

Variable	N (%)		χ2 (*p*)
	Self-Perceived Oral Health		
	Good	Poor	
Gingival bleeding			
Yes	138 (52.5)	125 (47.5)	12.663 (<0.001) ***
No	253 (66.4)	128 (33.6)	
Dry mouth			
Yes	125 (51.9)	116 (48.1)	12.638 (<0.001) ***
No	266 (66.0)	137 (34.0)	
Saliva viscosity			
Very Low	253 (63.3)	147 (36.8)	8.638 (0.035) *
Low	92 (61.3)	58 (38.7)	
High	39 (52.7)	35 (47.3)	
Very high	7 (35.0)	13 (65.0)	
Daily tooth brushing frequency			
1~2	97 (52.7)	87 (47.3)	6.922 (0.031) *
3	258 (64.0)	145 (36.0)	
≥4	36 (63.2)	21 (36.8)	
Smoking			
Yes	50 (56.2)	39 (43.8)	0.890 (0.345)
No	341 (61.4)	214 (38.6)	
Average weekly beverage consumption frequency			
None	83 (69.2)	37 (30.8)	9.482 (0.024) *
1~2	194 (62.8)	115 (37.2)	
3~4	86 (53.1)	76 (46.9)	
≥5	28 (52.8)	25 (47.2)	
Average daily sleeping duration			
≤5 h	127 (56.7)	97 (43.3)	4.594 (0.204)
≤6 h	153 (60.0)	102 (40.0)	
≤7 h	80 (67.8)	38 (32.2)	
≥8 h	31 (66.0)	16 (34.0)	

Note. * *p* < 0.05; *** *p* < 0.001.

**Table 2:** The relationship between subjective oral symptoms and lifestyle factors according to stress level.

Variable	N (%)				χ^2^ (*p*)
	Stress				
	Very Low	Low	High	Very High	
Gingival bleeding					15.130 (0.002) **
Yes	21 (8.0)	126 (47.9)	97 (36.9)	19 (7.2)	
No	55 (14.4)	210 (55.1)	98 (25.7)	18 (4.7)	
Dry mouth					8.771 (0.032) *
Yes	25 (10.4)	112 (46.5)	86 (35.7)	18 (7.5)	
No	51 (12.7)	224 (55.6)	109 (27.0)	19 (4.7)	
Saliva viscosity					25.939 (0.002) **
Very low	43 (10.8)	217 (54.3)	123 (30.8)	17 (4.3)	
Low	20 (13.3)	80 (53.3)	43 (28.7)	7 (4.7)	
High	13 (17.6)	31 (41.9)	22 (29.7)	8 (10.8)	
Very high	0 (0.0)	8 (40.0)	7 (35.0)	5 (25.0)	
Daily tooth brushing frequency					8.918 (0.178)
1~2	25 (13.6)	87 (47.3)	63 (34.2)	9 (4.9)	
3	47 (11.7)	218 (54.1)	117 (29.0)	21 (5.2)	
≥4	4 (7.0)	31 (54.4)	15 (26.3)	7 (12.3)	
Smoking					24.703 (<0.001) ***
Yes	9 (10.1)	37 (41.6)	28 (31.5)	15 (16.9)	
No	67 (12.1)	299 (53.9)	167 (30.1)	22 (4.0)	
Average weekly beverage consumption frequency					11.515 (0.242)
None	16 (13.3)	61 (50.8)	38 (31.7)	5 (4.2)	
1~2	38 (12.3)	172 (55.7)	83 (26.9)	16 (5.2)	
3~4	17 (10.5)	77 (47.5)	59 (36.4)	9 (5.6)	
≥5	5 (9.4)	26 (49.1)	15 (28.3)	7 (13.2)	
Average sleeping duration					18.297 (0.032) *
≤5 h	21 (9.4)	104 (46.4)	80 (35.7)	19 (8.5)	
≤6 h	30 (11.8)	136 (53.3)	78 (30.6)	11 (4.3)	
≤7 h	17 (14.4)	70 (59.3)	24 (20.3)	7 (5.9)	
≥8 h	8 (17.0)	26 (55.3)	13 (27.7)	0 (0.0)	

Note. * *p* < 0.05; ** *p* < 0.01; *** *p* < 0.001.

**Table 3:** Association Between Self-Perceived Oral Health and Tooth Brushing Frequency, Oral Symptoms, and Periodontal Disease Diagnosis.

Variable (Ref.)	Self-Perceived Oral Health (Ref. Poor)		
	Odds Ratio	95% Confidence Interval	*p*-Value
Average daily tooth brushing frequency	1.363	1.030–1.804	0.030 *
Periodontal disease (ref. No)	1.236	0.806–1.894	0.332
Dry mouth (ref. No)	0.576	0.413–0.804	0.001 **
Gingival bleeding (ref. No)	0.597	0.426–0.836	0.003 **

Note. * *p* < 0.05, ** *p* < 0.01.

**Table 4:** Coefficients among key survey items.

Variable	Stress Level	Gingival Bleeding	Dry Mouth	Saliva Viscosity	Daily Tooth Brushing Frequency	Smoking	Average Weekly Beverage Consumption Frequency	Average Daily Sleeping Duration
Stress level	1							
Gingival bleeding	0.148 **	1						
Dry mouth	0.106 **	0.056	1					
Saliva viscosity	0.078 *	0.108 **	0.252 **	1				
Daily tooth brushing frequency	−0.025	0.023	0.008	0.008	1			
Smoking	0.134 **	−0.040	0.006	0.080*	0.019	1		
Average weekly beverage consumption frequency	0.078 *	0.122 **	0.033	0.072	0.088 *	−0.045	1	
Average daily sleeping duration	0.143 **	0.000	0.055	0.051	−0.057	0.002	0.005	1

Note. * *p* < 0.05, ** *p* < 0.01.

## Data Availability

All data generated or analyzed during this study are included in this published article.
